# AIVIVE: a novel AI framework for enhanced *in vitro* to *in vivo* extrapolation (IVIVE) of toxicogenomics data

**DOI:** 10.1093/toxsci/kfaf100

**Published:** 2025-07-21

**Authors:** Mansi Chandra, Ting Li, Weida Tong

**Affiliations:** National Center for Toxicological Research, Food and Drug Administration, Jefferson, AR 72079, United States; University of Arkansas at Little Rock and University of Arkansas for Medical Sciences Joint Bioinformatics Program, Little Rock, AR 72204, United States; National Center for Toxicological Research, Food and Drug Administration, Jefferson, AR 72079, United States; National Center for Toxicological Research, Food and Drug Administration, Jefferson, AR 72079, United States

**Keywords:** generative AI, generative adversarial networks (GANs), toxicogenomics (TGx), local optimization, in vitro to in vivo extrapolation (IVIVE)

## Abstract

In vitro to in vivo extrapolation (IVIVE) of toxicogenomics (TGx) data is essential for enhancing mechanism-based toxicity evaluations and minimizing animal use. However, translating in vitro findings to in vivo responses remains challenging. Generative adversarial networks (GANs) show potential in synthesizing gene expression data but often miss subtle, toxicologically relevant signals. We developed **AIVIVE** (artificial intelligence-aided IVIVE), a novel framework integrating GANs with local optimizers guided by biologically relevant gene modules to improve prediction accuracy. AIVIVE was trained using rat liver in vitro and in vivo transcriptomic data from the Open TG-GATEs (Toxicogenomics Project–Genomics-Assisted Toxicity Evaluation System) database. AIVIVE was evaluated using cosine similarity, root mean squared error (RMSE), and mean absolute percentage error (MAPE), demonstrating synthetic profiles comparable to real biological replicates. Notably, the model showed high overlap with differentially expressed genes, including Cytochrome P450 enzymes, which are often underrepresented in vitro. AIVIVE recapitulated in vivo CYP expression patterns, overcoming in vitro limitations. Further analysis revealed that AIVIVE captured liver-related pathways like bile secretion, steroid hormone biosynthesis, hepatitis C, and chemical carcinogenesis. It also captured gene expression changes linked to liver-specific adverse outcome pathways, such as *Cyp2e1* upregulation in non-alcoholic fatty liver disease. Additionally, AIVIVE slightly outperformed real data in necrosis classification tasks, suggesting its potential for advancing toxicology predictions. These findings support AIVIVE as a tool for generating biologically relevant, in vivo-like profiles from in vitro data to enhance risk assessment, drug safety, and the 3Rs (reduce, replace, refine) principle.

Toxicogenomics (TGx) is an interdisciplinary field that integrates toxicology and genomics with bioinformatics to assess the toxic effects of compounds by analyzing their impact on gene expression (Chen et al. 2012; Liu et al. 2019b; Meier et al. 2025). This approach provides a comprehensive understanding of molecular toxicity mechanisms and has become essential in drug development for evaluating safety, predicting drug-induced toxicity, and identifying genomic biomarkers (Yang et al. 2004; Khor et al. 2006; Uehara et al. 2010; Chen et al. 2012; Khan et al. 2014). By uncovering toxicity pathways and early biomarkers, TGx enhances risk assessment, thereby improving chemical safety evaluations and protecting public health.

Nonetheless, TGx is expensive, with a high cost associated with data generation, collection, and analysis (Balbus 2005). Particularly, when TGx experiments are conducted on specific tissues or organs from animal studies, the cost is even higher due to animal use (Pettit et al. 2010; Vahle et al. 2018). Consequently, many TGx have been focused on in vitro platforms, which raises a question on how well in vitro TGx findings can be translated into those from animal models. This in vitro to in vivo extrapolation (IVIVE) method translates in vitro experimental findings into in vivo predictions (Liu et al. 2017), playing an important role in reducing reliance on animal use in risk and safety assessment (Liu et al. 2019a). By aligning with the 3R principles (Replacement, Reduction, and Refinement), this approach fosters the development of ethical and scientifically robust alternatives for toxicity assessments (Liu et al. 2019b; Avila et al. 2020; Lauwereyns et al. 2024; Poh and Stanslas 2024).

Recent advancements in artificial intelligence (AI), particularly generative AI (GenAI) techniques such as generative adversarial networks (GANs), have shown potential in generating large, high-dimensional synthetic data that closely mirrors real biological data. Their emergence as an innovative tool for synthesizing gene expression profiles has supported predictive modeling and biomarker discovery in genomics (Ahmed et al. 2021), as well as applications in toxicology and risk assessment (Sinha et al. 2023; Tan et al. 2024). We have developed specialized models such as ToxGAN, which generates synthetic TGx responses in rat liver based on chemical structure, dosage, and treatment time duration (Chen et al. 2022). GenAI also offers solutions for cross-domain extrapolation, such as text-to-image or text-to-video (Kim et al. 2020; Singh 2023). In other words, GenAI can generate synthetic data for one domain by learning the data from a different domain. We have evaluated GAN for cross-organ transcriptomic extrapolation with and without drug treatment (Li et al. 2023; Li et al. 2024).

The application of GANs in TGx studies comes with challenges. A characteristic challenge in TGx data is the low prevalence of genes exhibiting fold changes across experimental conditions, despite their involvement in key toxicological pathways. GANs often struggle to replicate these subtle expression patterns, potentially leading to the loss of important insights into toxicity mechanisms when mimicking real gene expression profiles. In this study, we proposed the inclusion of local optimizers (AI models) to improve the accuracy of these biologically relevant genes from the GAN output, with the aim of enhancing the accuracy of these genes, consequently enhancing the overall accuracy of synthetic data from GANs with a closer resemblance to real biological profiles. To demonstrate the effectiveness of this approach, we apply it to an IVIVE TGx study.

Specifically, we present AIVIVE (AI-aided IVIVE), a novel AI framework that enhances conventional GAN models with a local optimization approach using biologically relevant gene clusters (modules) (Callegaro et al. 2023) to improve the accuracy of synthetic transcriptomic profiles. We applied this framework to translate in vitro rat liver transcriptomic profiles from primary hepatocytes into in vivo (single-dose) profiles, using data from Open TG-GATEs (Toxicogenomics Project–Genomics-Assisted Toxicity Evaluation System) (Igarashi et al. 2015). AIVIVE’s performance was quantitatively assessed using cosine similarity, root mean squared error (RMSE), and mean absolute percentage error (MAPE). To assess the biological relevance of the generated profiles, we conducted a comparative analysis of differentially expressed genes (DEGs), enriched pathways, and adverse outcome pathway (AOP)-associated genes related to drug-induced liver toxicity. Additionally, we demonstrated the AIVIVE’s ability to predict drug-induced liver necrosis. Overall, AIVIVE was demonstrated as a robust framework for generating biologically meaningful synthetic transcriptomic profiles, with potential applications in risk assessment and safety evaluation of chemicals and drugs.

## Materials and methods

### TGx data from Open TG-GATEs

The rat liver in vitro and in vivo (single-dose) transcriptomic profiles and pathological findings were sourced from Open TG-GATEs (Igarashi et al. 2015), a comprehensive TGx database containing transcriptomic and toxicology data from primary rat hepatocytes (in vitro) and rat studies (in vivo). It includes compounds administered at 3 dose levels (low, middle, and high) along with controls across multiple time points, serving as a robust resource for toxicity assessment. For this study, we selected 3,350 in vitro and 6,671 in vivo (single-dose) rat liver transcriptomic profiles, covering 140 compounds common to both experiments. In vitro samples (with up to 2 biological replicates) were collected at 2, 8, and 24 h, whereas in vivo samples (with up to 5 biological replicates) were collected at 3, 6, 9, and 24 h. The transcriptomic profiles were retrieved from https://dbarchive.biosciencedbc.jp/en/open-tggates/download.html.

### Data preprocessing and train-test split

Transcriptomic files were normalized using the robust multi-array average method (Irizarry et al. 2003) via the “oligo” package (Carvalho and Irizarry 2010). Probe IDs were annotated using the rat2302.db database from the “AnnotationDbi” package (Pagès et al. 2021), and probes were filtered for the rat S1500+ gene set, which is relevant to toxicity studies under the Tox21 program and captures gene co-expression and common toxicity pathways (Mav et al. 2018). The final dataset contained 3,453 probes.

For each compound, pairwise samples were created by matching in vitro and in vivo transcriptomic profiles. Treatment groups were defined by compound, dose, and time and were paired only within the same compound. Control groups were processed similarly. Data were randomly split by compound into training (80%; 112 compounds) and test (20%; 28 compounds) sets, resulting in 79,398 training and 20,160 test pairwise profiles for AIVIVE model development and evaluation, respectively.

### AIVIVE model development and evaluation

The AIVIVE model was developed to perform IVIVE by translating transcriptomic profiles from in vitro to in vivo settings. It consisted of two components: A GAN-based translator that generated synthetic in vivo profiles based on in vitro profiles and a local optimization approach that employed multiple optimizers to refine low-signal values missed in the initial translation, improving the biological fidelity of the synthetic in vivo profiles.

#### GAN-based translator

The first component is a GAN-based translator with a structure similar to TransTox (Li et al. 2024), designed to translate in vitro transcriptomic profiles to in vivo using a generator-discriminator pair. The generator generated synthetic in vivo transcriptomic profiles from real in vitro data, and the discriminator evaluated both real and synthetic in vivo profiles, providing iterative feedback to refine the generator’s output. To enhance translation quality, a reverse in vivo-to-in vitro cycle assisted in the minimization of cycle loss for improved consistency, ensuring that the generated in vivo profiles retained biological relevance. The generator received a 6,938-dimensional input vector, which included a 3,453-dimensional real in vitro transcriptomic profile, two 16-dimensional binary vectors encoding source and target labels such as experiment type, dose, time, and biological replicates, as well as a 3,453-dimensional Gaussian noise vector drawn from a normal distribution. All input values were scaled to a range between 0 and 1 using a MinMax scaler. The generator was a fully connected neural network with 5 hidden layers containing 8,192, 7,168, 7,168, 4,096, and 4,096 nodes, respectively. A LeakyReLU activation function with an alpha value of 0.2 was applied to each layer except for the output layer, which consisted of 3,453 neurons corresponding to the dimensions of real in vivo transcriptomic profiles and used a sigmoid activation function. Dropout layers with rates of 0.8, 0.8, 0.8, and 0.4 were applied after each hidden layer except for the final one.

The discriminator was a fully connected neural network to classify input profiles as either real or synthetic. It had an input dimension of 3,453, corresponding to the feature dimensions of real or synthetic in vivo profiles. The network consisted of 2 hidden layers with 256 and 64 neurons, respectively, each activated by the ReLU function and followed by a dropout layer with a dropout rate of 0.5. The final output layer consisted of a single neuron with a sigmoid activation function for binary classification. The discriminator was trained using stochastic gradient descent with a learning rate of 0.0001 and a momentum of 0.9. Binary cross-entropy was employed as the loss function, given its effectiveness in optimizing models for binary classification tasks.

#### Local optimizers

The second component of AIVIVE included local optimizers to refine synthetic in vivo profiles by improving the expression of biologically important genes that were not appropriately simulated in the initial GAN-generated profiles. This process started with identifying DEGs in real in vivo profiles that were not adequately represented in the synthetic in vivo profiles within the training set. Subsequently, to guide biologically informed correction, we mapped the underrepresented DEGs to co-expression modules defined in the TXG-MAPr database (https://txg-mapr.eu/tg_rat_liver/) (Callegaro et al. 2023). Only modules containing at least 5 genes overlapping with the rat S1500+ gene set were selected to ensure sufficient biological relevance with an adequate amount of data for optimization. Thereafter, a neural network-based local optimizer was trained for each selected module using the training set. This process resulted in 21 local optimizers that were applied to the test set for generating the synthetic in vivo profiles.

#### Model evaluation

The performance of the AIVIVE model was quantitatively assessed on the test set using three widely recognized metrics: cosine similarity, RMSE, and MAPE. Cosine similarity measured the alignment between synthetic and real in vivo profiles, whereas RMSE quantified the average deviation, with lower values indicating higher model accuracy. MAPE provided a percentage-based measure of relative error, assessing how much the synthetic profiles deviated from real in vivo profiles. These metrics collectively offered a comprehensive evaluation of AIVIVE’s ability to generate biologically relevant synthetic data. Additionally, Welch’s two-sample *t*-test was applied to calculate *P*-values, determining the statistical significance of differences between groups (Welch 1947).

### DEGs, pathways, and AOPs gene analysis

In this study, we evaluated AIVIVE’s ability to preserve the biological relevance of synthetic in vivo transcriptomic profiles by comparing them to the real in vivo profiles with respect to DEGs, KEGG (Kyoto Encyclopedia of Genes and Genomes) pathway enrichment, and AOPs analysis.

#### Differentially expressed genes

DEGs were identified as those with an absolute fold change >1. For real transcriptomic profiles, fold change was calculated by comparing the averaged gene expression of the treatment group to its time-matched control, with gene expression values averaged across biological replicates. For AIVIVE-generated synthetic profiles, we followed a similar approach, where their averaged gene expression was compared with the real-time-matched control group.

In the current study design, each in vivo treatment condition typically consisted of 3 biological replicates. However, AIVIVE generated multiple synthetic triplet replicates of in vivo profiles using in vitro data with varying time and dose combinations under the same test compound. DEGs for each synthetic triplet were identified by comparing them to the corresponding in vivo control. The final DEG set for synthetic profiles within an in vivo treatment group was obtained as the union of these individual DEG sets.

The overlap between real and synthetic DEGs was analyzed by calculating the proportion of DEGs shared between both profiles, defined as the number of common DEGs divided by the total number of real DEGs. Treatments with no DEGs in real profiles were excluded to enable this calculation. The average DEG overlap ratio across treatments was assessed for each module and the overall rat S1500+ gene set.

#### KEGG pathways

The identified DEGs were mapped to KEGG (Kanehisa and Goto 2000) pathways using the clusterProfiler package (Yu et al. 2012) to determine enriched pathways, applying a Bonferroni-corrected *P*-value cutoff of ≤0.05. Enriched KEGG pathways were identified for both real and synthetic in vivo profiles, and their overlap was compared.

#### AOPs genes

We also conducted a comparative analysis focusing on AOPs genes related to drug-induced liver toxicity (Khadka et al. 2020). Genes overlapping between AOPs and the rat S1500+ gene set were selected for comparison. The real and synthetic in vivo profiles were assessed by calculating the percentage error in gene expression for test set compounds, specifically targeting high-dose and 24-h treatment groups. This analysis was essential for determining the AIVIVE’s ability to replicate gene expression patterns relevant to toxicity pathways. The percentage error between real and synthetic profiles was calculated using Formula 1.


(1) $$\mathrm{Error}\;(Gene\;\mathrm{Expression})\%=\;\frac{\left(\mathrm{Real}-\mathrm{Synthetic}\right)}{\mathrm{Real}\;}\;\times \;100\%$$

### Necrosis prediction

We evaluated the AIVIVE-generated transcriptomic profiles for predicting necrosis, a common pathological feature of drug-induced liver injury (DILI) (Iorga et al. 2017). Treatments were classified as “Necrosis Positive” if at least 1 sample exhibited necrosis and “Necrosis Negative” if no pathological findings were observed across all samples, which resulted in 76 necrosis-positive and 990 necrosis-negative treatments in the in vivo training set. To balance the dataset for necrosis classification, 76 necrosis-negative treatments were randomly selected and paired with the necrosis-positive treatments. Gene expression values for each treatment were averaged across biological replicates for necrosis model development. An XGBoost classifier model was then developed with the following parameters: N _estimators = 100, learning_rate = 0.1, and max_depth = 10.

The trained XGBoost model was evaluated on 2 test sets: Real transcriptomic profiles and synthetic transcriptomic profiles generated by AIVIVE. Both test sets consisted of 16 necrosis-positives and 244 necrosis-negatives. Model performance was assessed using accuracy, measuring the proportion of correct predictions (true positives and true negatives) relative to all predictions. This evaluation provided a comprehensive comparison of model performance across both real and synthetic datasets.

## Results

### AIVIVE model development and performance

**Fig. 1.**
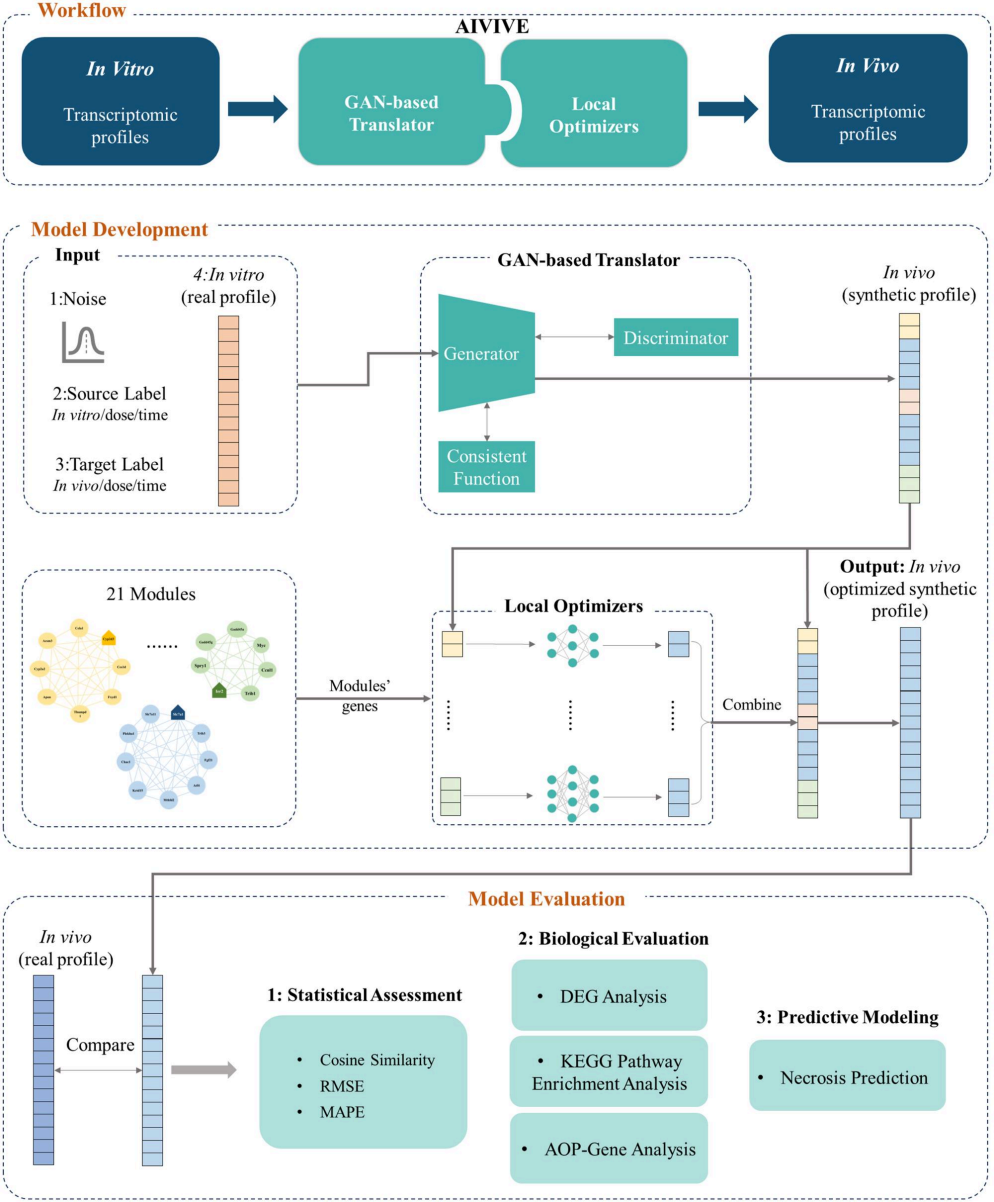
AIVIVE architecture. Workflow: The flowchart depicts the workflow of in vitro to in vivo extrapolation (IVIVE) using AIVIVE. Model development: The AIVIVE framework integrates a GAN-based translator with local optimizers. The translator consists of a generator, a discriminator, and a consistency function leveraging cycle loss to enhance synthetic profile fidelity, enabling effective IVIVE. The generator takes a concatenated input, including the real in vitro transcriptomic profile, source label information (in vitro/dose/time), target label information (in vivo/dose/time), and a noise vector. The discriminator distinguishes synthetic from real in vivo profiles and provides feedback to the generator, enhancing the quality of synthetic profile generation. Local optimizers refine the synthetic profiles by optimizing gene clusters with biological relevance to generate more accurate in vivo representations. There were 21 modules that were optimized. Model evaluation: The AIVIVE model was assessed on the test set, and its capabilities were demonstrated through various analyses, including statistical assessment (cosine similarity, RMSE, and MAPE), biological evaluation (DEG analysis, KEGG pathway enrichment analysis, and AOPs-gene analysis), and predictive modeling (necrosis prediction).

As depicted in Fig. 1, AIVIVE is a two-component model to generate high-quality synthetic in vivo transcriptomic profiles. The first component, a GAN-based translator, employs a similar structure as TransTox (Li et al. 2024) to transform in vitro transcriptomic profiles into in vivo profiles. It was trained on 79,398 in vitro-in vivo profile pairs from 112 compounds in the Open TG-GATEs database (Igarashi et al. 2015). The optimal translator was selected at epoch 3,717, where the loss function had stabilized.

The second component, a local optimization approach, zoomed in on the biologically relevant genes that were likely missed by the GAN model. Specifically, we focused on gene modules—clusters of co-regulated genes identified through Weighted Gene Co-expression Network Analysis (WGCNA) (Langfelder and Horvath 2008) that represent biologically meaningful pathways or functions. Callegaro et al. report a list of gene modules consistent across in vitro and in vivo data from TG-GATEs (Callegaro et al. 2023). We employed these modules to enhance synthetic in vivo profiles using local optimizers. Following predefined selection criteria (see Materials and methods Section), we identified 27 modules, each named after its hub gene—the most highly connected gene within the module. Of these, 21 modules showed improved performance compared with the GAN results (Fig. S1). Each of the 21 modules comprised a unique set of genes, with gene counts ranging from 5 to 41, totaling 239 genes across 345 probes. The final AIVIVE model integrated the GAN model with the 21 local optimizers corresponding to these modules.

**Fig. 2.**
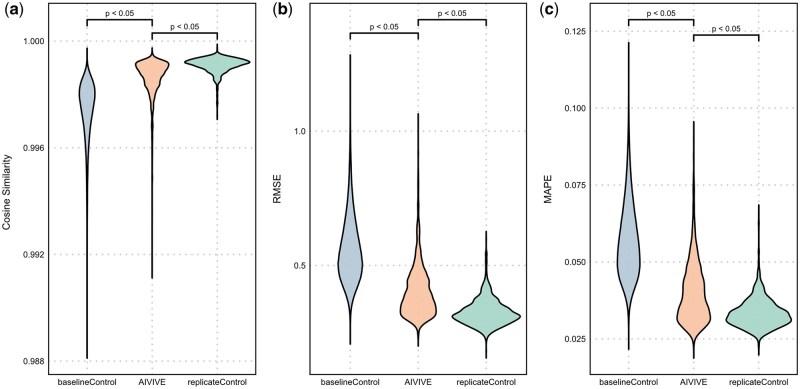
Evaluation of AIVIVE performance on test set. The performance of AIVIVE was assessed on the test set from TG-GATEs. Three metrices were used for evaluating model performance: cosine similarity, RMSE, and MAPE (a to c). The baseline control group depicts measurements between any 2 real in vivo profiles (excluding biological replicates). The AIVIVE group includes measurements between the optimized synthetic in vivo profiles and their analogous real ones. The replicate control group refers to the comparisons between the biological replicates within a treatment.

We applied AIVIVE to the test set which consisted of profiles from 28 independent compounds (20,160 paired profiles). We quantitatively evaluated AIVIVE’s performance on the test set using cosine similarity, RMSE, and MAPE (Fig. 2). To better contextualize the results, we compared 3 groups: AIVIVE, a baseline control, and a replicate control. The AIVIVE group assessed the similarity between synthetic profiles and their corresponding real profiles. The baseline control measured the concordance (e.g. correlation or distance) between pairs of real in vivo profiles from different treatments, serving as a reference point that AIVIVE should exceed. The replicate control, on the other hand, measured the performance between biological replicates within the same treatment, serving as a reference point that AIVIVE may achieve if the synthetic data are considered as biological replicates.

The AIVIVE group consistently outperformed the baseline control, with significantly higher cosine similarity (0.9986 vs. 0.9975), and lower RMSE (0.404 vs. 0.545) and MAPE (0.0399 vs. 0.0558) with *P* < 0.05. Although the AIVIVE group showed slightly lower performance than the replicate control (cosine similarity: 0.9986 vs. 0.9991, RMSE: 0.404 vs. 0.3264, MAPE: 0.0399 vs. 0.0334) (*P* < 0.05), it was still notably closer to the replicate control than to the baseline control across all statistical measures. Overall, these results demonstrate that AIVIVE effectively captures in vivo gene expression trends, generating synthetic profiles that closely resemble real biological replicates.

### AIVIVE applications: DEGs, pathways, and AOPs gene analyses

To assess the utility of synthetic in vivo transcriptomic profiles generated by AIVIVE, we first evaluated DEGs, and the extension of the pathways derived from DEGs, in comparison with the real profiles. Because both the data used were related to DILI, we then compared the synthetic and real profiles with respect to the DILI-related AOPs genes. All the analyses were carried out on the test set.

#### Differentially expressed genes

DEGs were identified from both real and synthetic in vivo profiles within the test set, and the average overlap ratio between the two was calculated (see Materials and methods Section). Our analysis revealed that the DEG overlap ratio was 48%. Of note, TransTox (Li et al. 2024) previously reported an average DEG overlap ratio of approximately 40% between real experimental groups generated by the same laboratory, suggesting that the AIVIVE-generated synthetic profiles achieved a comparable level of agreement to real experimental data.

We also zoomed into Cytochrome P450 (CYP) genes that play a key role in drug metabolism, detoxification, and xenobiotic processing in the liver (Martignoni et al. 2006; Guo et al. 2014; Hammer et al. 2021). Although CYP genes are known to be highly active in vivo, their expression is often reduced or absent in vitro due to the loss of liver-specific functions in hepatocyte cultures over time (Turpeinen et al. 2009; Soldatow et al. 2013). Thus, we specifically focused on those treatments that showed no differential expression of CYP genes in real in vitro profiles but exhibited CYP expression in real in vivo profiles to examine whether the AIVIVE-generated synthetic profiles from in vitro profiles still reflect real in vivo biological mechanisms. The analysis was centered on the 24-h treatment groups, the only common time point between in vitro and in vivo experiments. Among the 21 treatments with no CYP expression in vitro, 20 exhibited CYP expression in real in vivo profiles. Further analysis revealed that AIVIVE successfully captured CYP expression trends in 17 of these 20 treatments, aligning closely with real in vivo profiles. Table 1 summarizes the number of differentially expressed CYP genes across these 20 treatments in both real and synthetic profiles.

**Table 1.** CYP gene expression in high dose and 24-h treatment groups (Test set).

Treatments (test set)	In vitro	In vivo (real)	In vivo (synthetic)
Benzbromarone_24 hr_Low	0	4	2
Allopurinol_24 hr _Low	0	2	1
Allopurinol_24 hr_Middle	0	2	1
Allopurinol_24 hr_High	0	4	3
Thioridazine_24 hr_Low	0	2	0
Flutamide_24 hr_Low	0	2	0
Chlorpheniramine_24 hr_Low	0	1	1
TNFÉØ_24 hr_Low	0	1	1
TNFÉØ_24 hr_Middle	0	2	2
Clomipramine_24 hr_Low	0	2	2
Lornoxicam_24 hr_Low	0	1	1
Lornoxicam_24 hr_High	0	6	1
Chlormadinone_24 hr_Low	0	1	0
Danazol_24 hr_Low	0	3	1
Danazol_24 hr_Middle	0	8	5
Cephalothin_24 hr_Low	0	4	1
Cyclosporine A_24 hr_Low	0	3	1
Aspirin_24 hr_Low	0	4	1
Amiodarone_24 hr_Low	0	8	3
Amiodarone_24 hr_Middle	0	6	1

#### KEGG pathway enrichment analysis

**Fig. 3.**
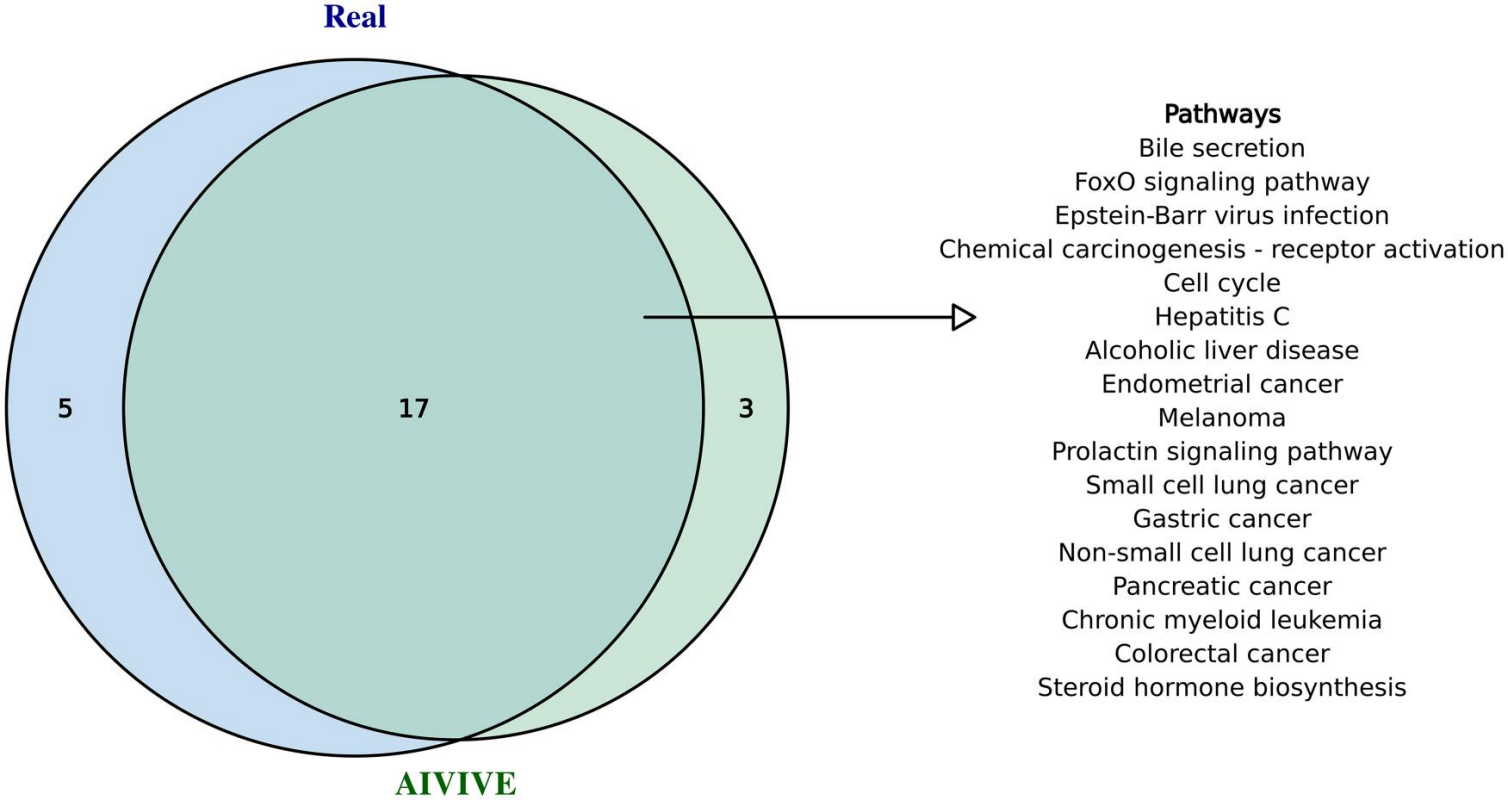
KEGG pathways enrichment. DEGs were mapped to KEGG pathways to assess pathway enrichment in both real in vivo transcriptomic profiles and optimized synthetic in vivo profiles from the test set. This figure presents a Venn diagram illustrating the top 20 KEGG pathways enriched in both real and optimized synthetic profiles. The pathways listed in the figure represent the shared pathways between the real and optimized synthetic profiles among the top 20 pathways analyzed.

KEGG pathway enrichment analysis was independently conducted on DEGs derived from real and AIVIVE-generated synthetic profiles. Comparison of the enriched pathways revealed a 36% overlap between the two, indicating an adequate level of concordance. This result is consistent with pathway overlap ratios observed among real experimental groups, as previously reported by TransTox (Li et al. 2024). To further validate our findings, we analyzed the top 20 enriched pathways in both real and AIVIVE-generated synthetic profiles, and their overlap was assessed to highlight the common pathways associated with liver toxicity and related mechanisms, as shown in Fig. 3. Due to identical ranking frequencies, 22 pathways were included in the real in vivo profile. Among these, 17 pathways overlapped between real and optimized synthetic profiles, demonstrating that AIVIVE effectively captured liver toxicity mechanisms and related biological processes observed in real in vivo data.

#### DILI AOPs gene analysis

**Fig. 4.**
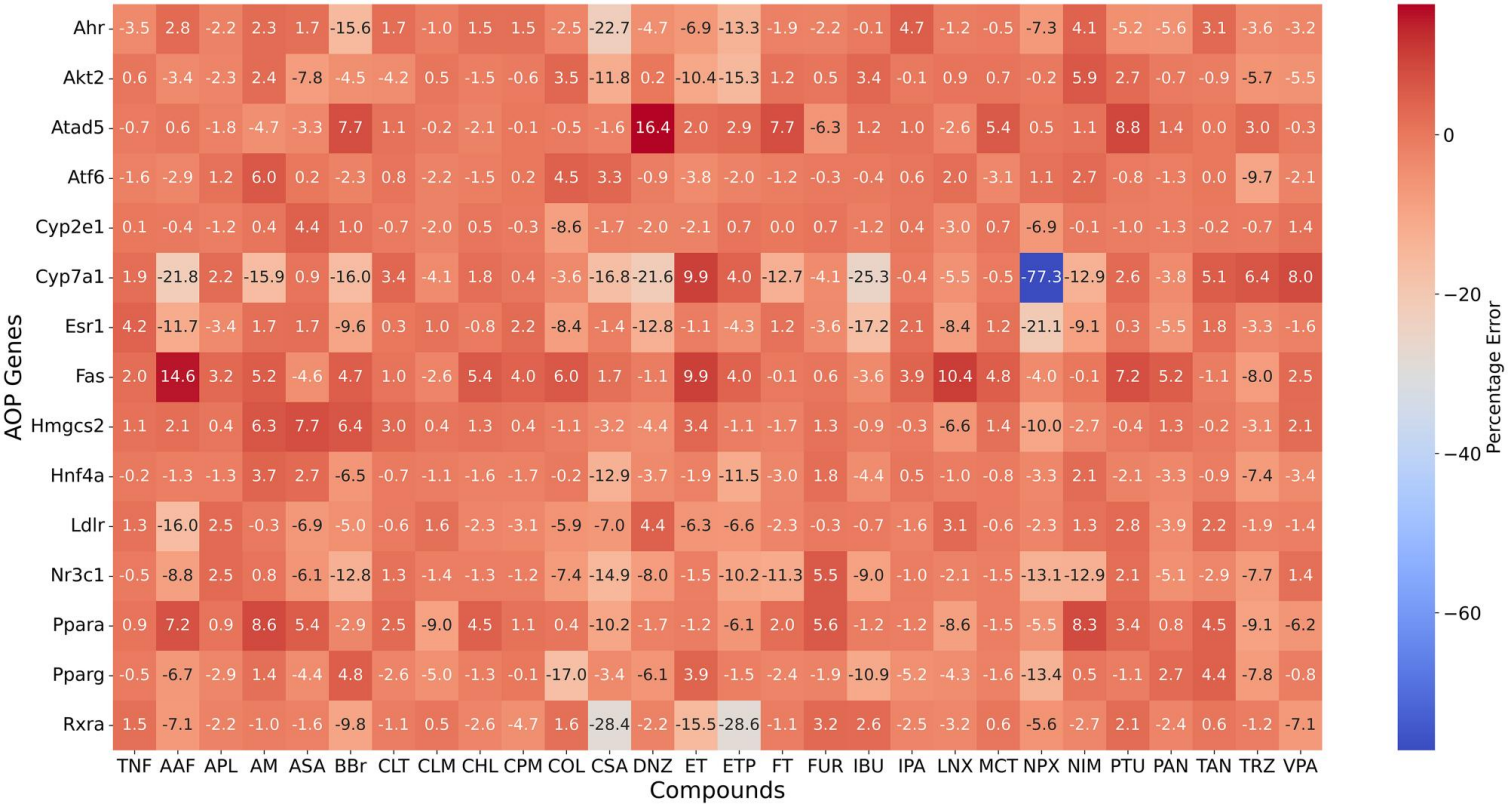
Comparison of gene expression patterns of AOP-related genes in real and AIVIVE-generated profiles. AOP-related genes were analyzed to study the concordance in their expression patterns and evaluate the performance of AIVIVE-generated profiles. 15 AOP-related genes, identified as part of the rat S1500+ gene set used in the development of AIVIVE, were analyzed for their expression across 28 compounds in the test set. The heatmap displays the percentage error in gene expression values between the real and optimized synthetic profiles for these 15 AOP-related genes. The *x*-axis represents the 28 test compounds, whereas the *y*-axis lists the 15 AOP genes. The color intensity indicates the magnitude of the percentage error, highlighting the patterns in gene expression between the real and optimized synthetic profiles across different compounds. TNF-α, tumor necrosis factor-alpha; AF, acetamidofluorene; APL, allopurinol; AM, amiodarone; ASA, aspirin; BBr, benzbromarone; CLT, cephalothin; CLM, chlormadinone; CHL, chlorpheniramine; CPM, clomipramine; COL, colchicine; CSA, cyclosporine A; DNZ, danazol; ET, ethionine; ETP, etoposide; FT, flutamide; FUR, furosemide; IBU, ibuprofen; IPA, iproniazid; LNX, lornoxicam; MCT, monocrotaline; NPX, naproxen; NIM, nimesulide; PTU, propylthiouracil; PAN, puromycin aminonucleoside; TAN, tannic acid; TRZ, thioridazine; VPA, valproic acid.

Because both in vitro and in vivo studies from TG-GATEs are designed for DILI study, we identified the DILI genes based on DILI AOPs. AOPs have become integral to toxicology research, serving as a framework to connect molecular mechanisms with toxicity events while supporting environmental risk assessment (Perkins et al. 2015; Saarimäki et al. 2023). In this study, we curated AOP-related genes from Khadka et al. (2020), who demonstrated their potential for predicting DILI. From their findings, we identified 15 unique genes that overlapped with the rat S1500+ gene set, enabling their use as exemplars for evaluating AIVIVE performance. To evaluate the alignment between real and AIVIVE-generated synthetic profiles, we calculated the percentage error in gene expression values of these 15 genes in the test set compounds under high-dose and 24-h treatment conditions. Figure 4 presents the percentage error in gene expression values between the real and synthetic profiles for the 28 test set compounds. The results showed that 309 out of 420 observations (73.57%) had a percentage error of 5% or less, indicating that the optimized synthetic profiles closely mirrored real in vivo profiles and effectively captured DILI-related gene expression patterns, where *Cyp2e1* exhibited the lowest average error, whereas *Cyp7a1* showed the highest.

### Necrosis prediction

We assessed the AIVIVE-generated transcriptomic profile for predicting necrosis, a key pathological event in drug-induced liver toxicity (Iorga et al. 2017). To achieve this, we developed an XGBoost classification model that was trained on 152 real in vivo profiles and evaluated on two test sets: one comprising real in vivo profiles and the other consisting of optimized synthetic in vivo profiles generated by AIVIVE. Both test sets included 16 necrosis-positive and 244 necrosis-negative samples.

**Fig. 5.**
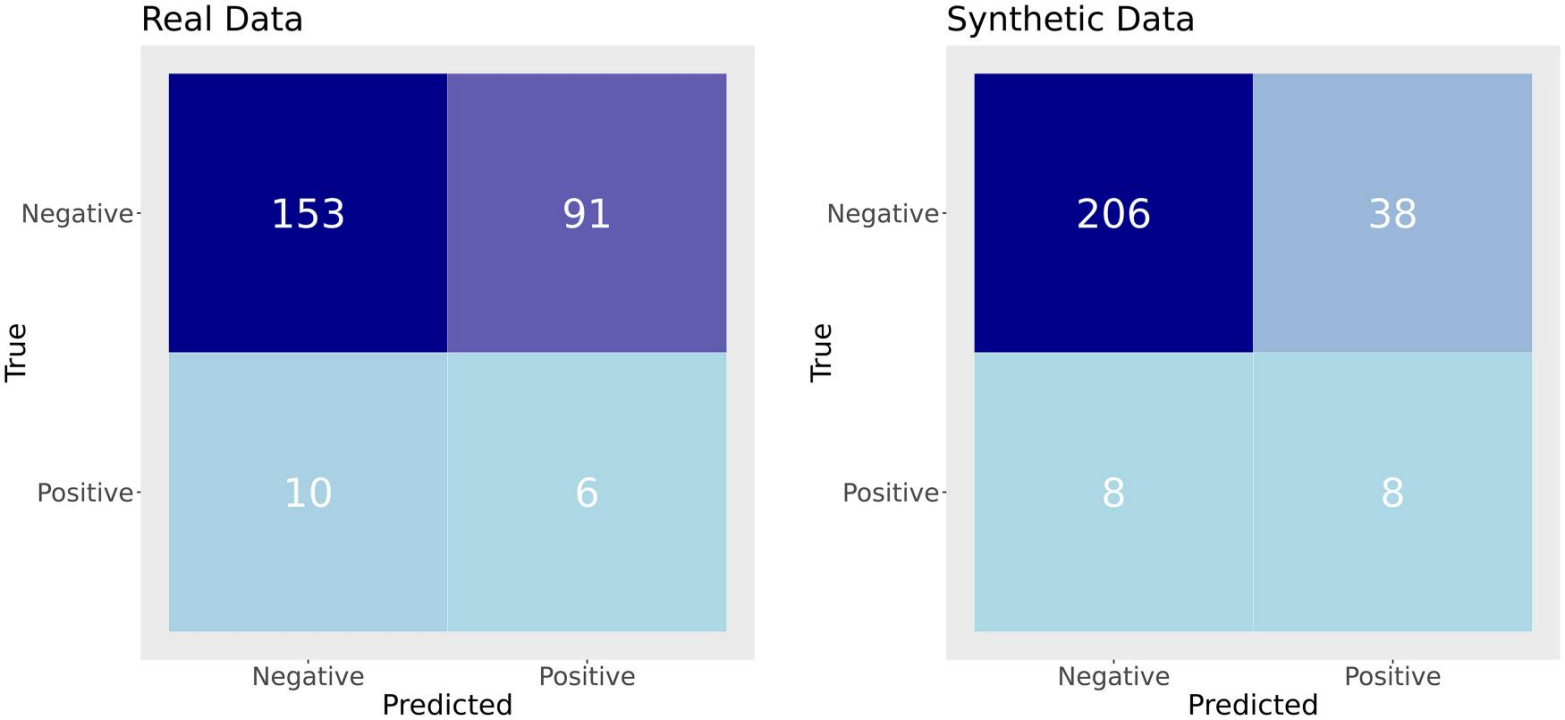
Performance of necrosis predictive model. The performance of the necrosis predictive model was assessed using both real and synthetic transcriptomic profiles from the test sets. The confusion matrix on the left shows the evaluation of the model’s predictions on real in vivo transcriptomic profiles, with the following values: true positives (TP) = 6, true negatives (TN) = 153, false positives (FP) = 91, and false negatives (FN) = 10. On the right, the confusion matrix shows the evaluation of the model’s performance on synthetic in vivo profiles generated by AIVIVE, with the following values: TP = 8, TN = 206, FP = 38, and FN = 8.

The confusion matrix in Fig. 5 illustrates the necrosis model’s performance across both test sets. Notably, the synthetic profile test set demonstrated better performance, achieving a higher number of true positives and true negatives compared with the real profile test set. The overall classification accuracy further supported this trend, with the model yielding an accuracy of 61.15% on the real in vivo test set and 82.31% on the synthetic in vivo test set, highlighting the potential of AIVIVE-generated profiles in predictive toxicology.

## Discussion

TGx has been an important part of drug discovery and development in evaluating drug-related risk factors, including mechanisms of action, dose-related toxicity, and biomarkers (Castle et al. 2002; Suter et al. 2004; Meier et al. 2025). The arrival of advanced AI methodologies, such as deep learning and GenAI, have generated an opportunity to enhance TGx application (Chen et al. 2022; Lin and Chou 2022; Li et al. 2023, 2024). A key challenge in using GenAI, such as GANs, for synthetic gene expression profiling is their inability to accurately simulate the complexity of real biological data, particularly the biologically important genes with low prevelance of differential expression. In this study, we present AIVIVE, a novel AI framework that combines a GAN-based translator with a local optimization approach by emphasizing on biologically meaningful genes for improved accuracy of synthetic transcriptomic data. The accuracy of synthetic results was evaluated on the test set. The findings demonstrated the robustness of AIVIVE and its potential for advancing TGx-based research in reducing the reliance on animal use to support toxicity risk assessments and predictive modeling.

First, the performance of AIVIVE was assessed using three metrics: cosine similarity, RMSE, and MAPE. These statistical measures assess the synthetic profiles’ accuracy in replicating the real profiles. The synthetic profiles were not only significantly higher than random chance in comparison to the baseline controls but also much closer to the real biological replicates (compared with the replicate control), highlighting AIVIVE’s ability to generate synthetic profiles comparable to the real experiments. This was further demonstrated with high DEG overlap ratios between real and synthetic profiles. Importantly, AIVIVE effectively captures in vivo-specific mechanisms, as evidenced by its ability to identify differentially expressed CYP genes. CYP enzymes, a superfamily of heme-containing proteins primarily found in the liver, play a crucial role in the metabolic detoxification of drugs, xenobiotics, and other biochemicals (Pustylnyak et al. 2007). These enzymes facilitate biotransformation, converting substances into metabolites, some of which become reactive and contribute to DILI (Guillouzo and Guguen-Guillouzo 2008; Corsini and Bortolini 2013). However, in vitro hepatocyte cultures often exhibit significant variability in CYP activity and fail to express many key CYP enzymes involved in drug metabolism, with approximately 70% of Cytochrome P450 content lost within the first 24 h of culture (Padgham and Paine 1993; Akrawi et al. 1994; Guillouzo and Guguen-Guillouzo 2008; Turpeinen et al. 2009; Soldatow et al. 2013). The synthetic in vivo profiles generated by AIVIVE successfully captured CYP gene expression patterns observed in real in vivo profiles, even in cases where CYP expression was absent in in vitro experiments. These results implied that AIVIVE might be able to address limitations of in vitro systems, such as the progressive loss of crucial liver metabolic functions, by generating synthetic transcriptomic profiles that more accurately reflect in vivo conditions.

We specifically evaluated the synthetic data accuracy to capture key biological pathways. For example, the synthetic profiles successfully reflected essential processes, including bile secretion (Arab et al. 2017; Chiang 2017), hepatitis C (Schuppan et al. 2003), alcoholic liver disease (Walsh and Alexander 2000; Tilg et al. 2011), steroid hormone biosynthesis (Yang et al. 2021), and chemical carcinogenesis (Karin and Dhar 2016)—all of which are fundamental to liver metabolism. Dysregulation of these pathways has been linked to liver disorders, with impaired bile secretion contributing to cholestasis (Trauner et al. 1998) and non-alcoholic fatty liver disease (NAFLD) (Jiao et al. 2018), whereas hepatitis C virus is a major driver of liver fibrosis, steatosis, hepatocellular carcinoma, and liver failure (Choi and Ou 2006; Yamane et al. 2013). Furthermore, we analyzed genes associated with liver AOPs for AIVIVE’s ability to capture gene expression patterns associated with liver toxicity. We found that, e.g. AIVIVE effectively replicated the expression pattern of *Cyp2e1* (Cytochrome P450-2E1), a hepatic enzyme responsible for the biotransformation of substances like ethanol and upregulated in NAFLD (Leung and Nieto 2013; Abdelmegeed et al. 2017).

We also assessed the value of synthetic profiles in predictive modeling for necrosis and found that the synthetic profiles generated by AIVIVE demonstrated better classification performance compared with real profiles. These results suggest that AIVIVE is a valuable tool for data generation, and its synthetic profiles could enhance predictive modeling in toxicological research.

AIVIVE incorporates a local optimization strategy based on biologically meaningful gene modules to improve the generation of synthetic transcriptomic profiles. This strategy enhances the AIVIVE’s ability to simulate gene expression patterns that are critical for biological responses. We utilized 21 biologically relevant gene modules previously derived from the TG-GATEs dataset (Callegaro et al. 2023) using WGCNA (Langfelder and Horvath 2008). WGCNA is a widely applied method in TGx and has been used for identifying cancer biomarkers (Hu et al. 2020; Tian et al. 2020), assessing DILI (Sutherland et al. 2018), and elucidating gene–phenotype relationships in the TG-GATEs dataset (Callegaro et al. 2023). The overlap of each module with the S1500+ gene set ranged from 5 to 41 genes.

To evaluate the impact of the local optimization, we compared DEG overlap between real and synthetic profiles across all modules, with and without the use of local optimizers. DEG overlap improved consistently across all modules when local optimization was applied (Fig. S1). A similar trend was observed in pathway enrichment analyses, where pathway overlap between real and synthetic data was also enhanced by local optimization (Fig. S2). It is noteworthy that the 21 gene modules used in this study were non-overlapping, each representing distinct biological processes. We opted to incorporate all modules into AIVIVE, as optimizing across multiple gene modules—rather than a single one—enables broader biological coverage and improves the accuracy of the generated in vivo transcriptomic profiles. To evaluate this, we compared DEG overlap ratios between real and synthetic profiles under three conditions: (i) without the local optimizer, (ii) using the best-performing single module, and (iii) using all 21 modules (Fig. S3). Results indicated that incorporating all biologically relevant modules significantly enhanced the biological fidelity of the synthetic data, demonstrating improved performance in IVIVE applications.

To assess the effectiveness of the AIVIVE model in extrapolating in vitro data to in vivo profiles for DEG and KEGG pathway enrichment analyses, we compared these metrics between AIVIVE-generated profiles and real in vivo data, using direct in vitro to in vivo comparisons under matched treatment conditions as a baseline. As shown in Fig. S4, the overlap of DEGs and enriched KEGG pathways is consistently lower in the baseline comparison than in the AIVIVE-extrapolated profiles. This discrepancy underscores the limitations of in vitro data in capturing the biological complexity of in vivo systems. By leveraging AIVIVE to bridge this gap, we achieve more accurate predictions of in vivo-like gene expression patterns and associated biological pathways.

Collectively, these findings demonstrate the value of AIVIVE’s module-based optimization in improving biological relevance, with potential reduction of animal use in toxicological studies in alignment with the 3Rs principle (Replacement, Reduction, and Refinement) (Liu et al. 2019b; Avila et al. 2020; Chang et al. 2022; Poh and Stanslas 2024). However, it is worth noting that, due to the limited number of chemicals in our dataset, we are currently unable to assess the performance of AIVIVE in new or distinct chemical spaces. In addition, not all liver-specific genes were accurately simulated by AIVIVE. For example, the expression of *Cyp7a1*, which encodes cholesterol 7α-hydroxylase and is regulated by bile acid signaling, was less accurately simulated (Zhang et al. 2009). Looking ahead, several directions could further refine and expand the AIVIVE model. Future developments may focus on enhancing its ability to capture a broader spectrum of biological processes by integrating diverse datasets (such as a wider range of chemical structures) and employing advanced training methodologies. Expanding the dataset to include a wider range of chemical structures will be essential for improving the model’s generalizability to novel compounds. Additionally, improving the AIVIVE’s sensitivity to treatment-specific factors, such as compound type, dose, and exposure duration, would further enhance its applicability to TGx studies.

The integration of a GAN-based translator with a local optimization approach utilizing biologically relevant modules represents an advancement in applying GenAI methods such as GANs in TGx research. This innovative approach provides a distinct advantage in modeling complex treatment-dependent gene expression patterns, addressing challenges that traditional methods struggle to replicate. In the long term, AIVIVE has the potential to become a powerful tool in both drug safety and TGx research. By offering molecular insights into the toxicity mechanisms with synthetic data, AIVIVE may be a promising alternative for efficient, ethical, and reliable toxicity risk assessments, ultimately advancing drug safety.

## Supplementary Material

kfaf100_Supplementary_Data

## Data Availability

The datasets used to develop and evaluate AIVIVE are curated from the Open TG-GATEs database available for download at https://dbarchive.biosciencedbc.jp/en/open-tggates/download.html. The modules were identified and downloaded from TXG-MAPr platform https://txg-mapr.eu/WGCNA_liver/TGGATEs_LIVER/. The training and test prediction files have been deposited at Zenodo under https://doi.org/10.5281/zenodo.14984579.

## Code availability

AIVIVE was developed using tensorflow-GPU version 2.4.1 with open-source Python (version 3.11.7). The RMA normalization and KEGG pathway analysis were carried out in R (version 4.4.1). The source code is available at https://github.com/CHANDMX20/AIVIVE.
