# Supplementary material for: AIVIVE: a novel AI framework for enhanced in vitro to in vivo extrapolation (IVIVE) of toxicogenomics data
Source: Toxicol Sci. 2025 Jul 21;207(2):361–71. doi: 10.1093/toxsci/kfaf100 (PMC12469192; doi:10.1093/toxsci/kfaf100)
Supplement: kfaf100_Supplementary_Data [file kfaf100_supplementary_data.docx]

**AIVIVE: A Novel AI Framework for Enhanced *In Vitro* to *In Vivo* Extrapolation (IVIVE) of Toxicogenomics Data**

**Mansi Chandra^1,2^, Ting Li^1,^ *, and Weida Tong^1^**

**^1^ National Center for Toxicological Research, Food and Drug Administration, Jefferson, Arkansas 72079, USA.**

**^2^ University of Arkansas at Little Rock and University of Arkansas for Medical Sciences Joint Bioinformatics Program, Little Rock, Arkansas 72204, USA**

*** Corresponding author: Ting Li (**[**Ting.Li@fda.hhs.gov**](mailto:Ting.Li@fda.hhs.gov)**)**


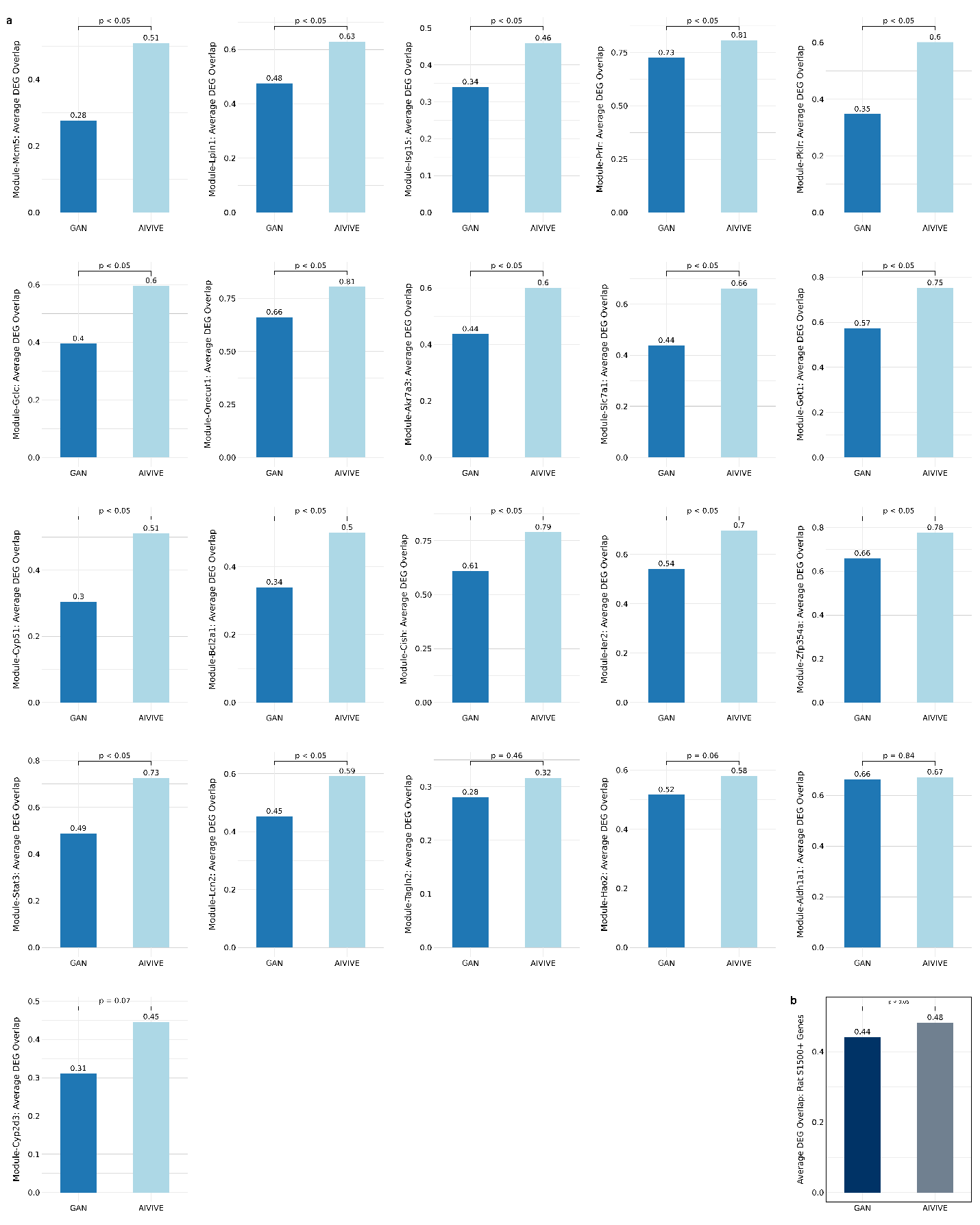


**Supplemental Figure 1:** *Comparison of DEG Overlap: GAN- Based Translator vs AIVIVE*

DEGs were identified in real *in vivo* transcriptomic profiles, as well as in both non-optimized and optimized synthetic *in vivo* profiles from the test set. The bar plots display the average DEG overlap ratio between real and non-optimized synthetic profiles, compared to real and optimized synthetic profiles. Panel **1a** displays the average DEG overlap ratios for the 21 modules optimized during the optimization process, while panel **1b** shows the overlap for the rat S1500+ gene set. In both panels, the x-axis represents two groups: 'GAN,' which indicates the overlap between non-optimized synthetic profiles and real profiles, and 'AIVIVE,' which represents the overlap between optimized synthetic profiles and real profiles. The y-axis represents the average DEG overlap ratio.


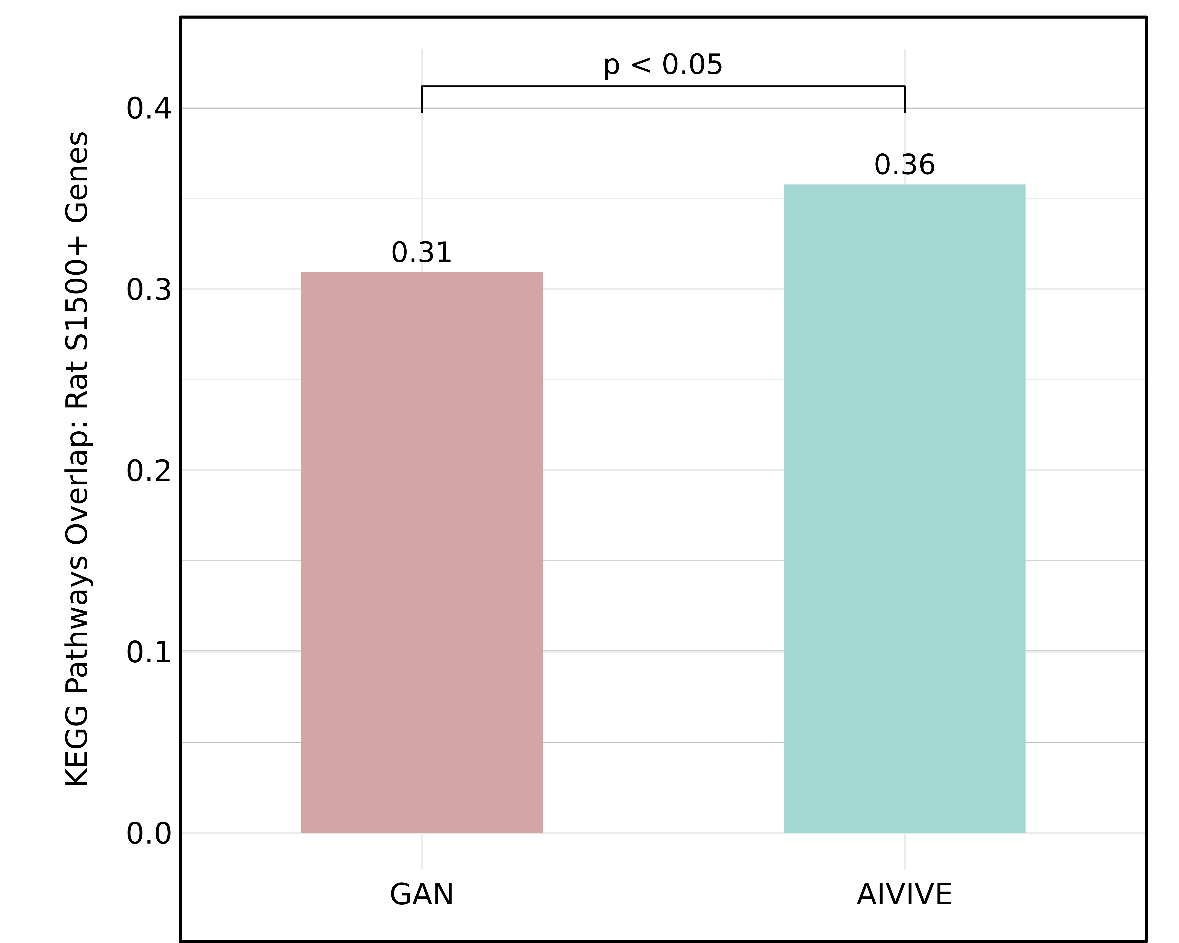


**Supplemental Figure 2:** *Comparison of KEGG Pathway Overlap: GAN-Based Translator vs AIVIVE*

The enriched KEGG pathways were analyzed in real *in vivo* transcriptomic profiles, as well as in both non-optimized and optimized synthetic *in vivo* profiles from the test set. The bar plots illustrate the average KEGG pathway overlap ratio between real and non-optimized synthetic profiles, compared to real and optimized synthetic profiles. This figure shows the average KEGG pathway overlap ratios for the DEGs identified in the rat S1500+ gene set. The x-axis represents the two groups: 'GAN,' indicating the overlap between non-optimized synthetic profiles and real profiles, and 'AIVIVE', representing the overlap between optimized synthetic profiles and real profiles. The y-axis shows the average KEGG pathway overlap ratio.


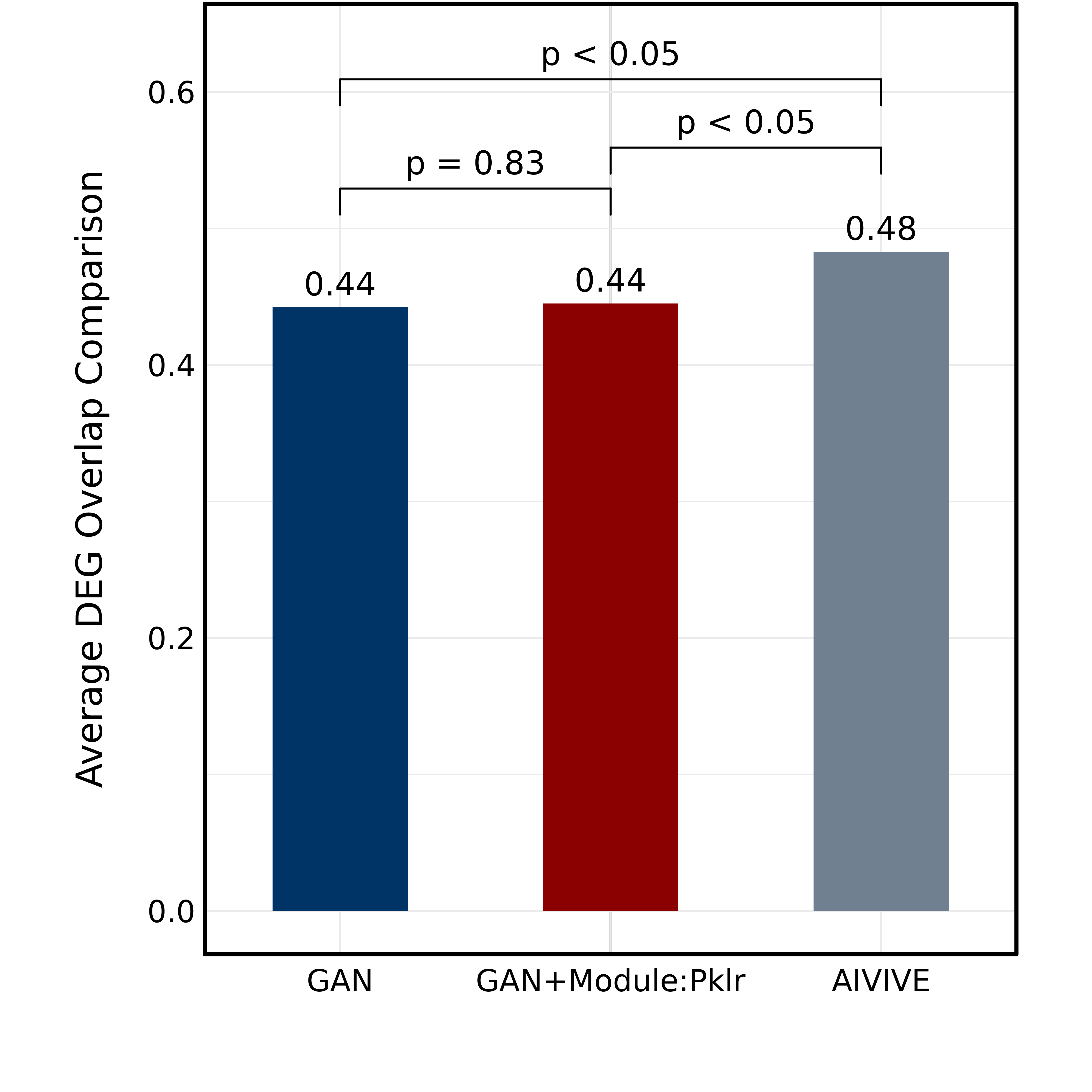


**Supplemental Figure 3:** *Comparison of DEG Overlap: GAN vs. GAN+Single Module vs. AIVIVE (GAN+21 Modules)*

DEGs were identified in real *in vivo* profiles and in synthetic profiles generated by the GAN-based translator under three conditions: without module optimization (GAN), with a single module (GAN+Module:Pklr), and with all 21 biologically meaningful modules (AIVIVE). The bar plot shows the average DEG overlap ratio between synthetic and real *in vivo* profiles, illustrating that multi-module optimization in AIVIVE improves biological fidelity.


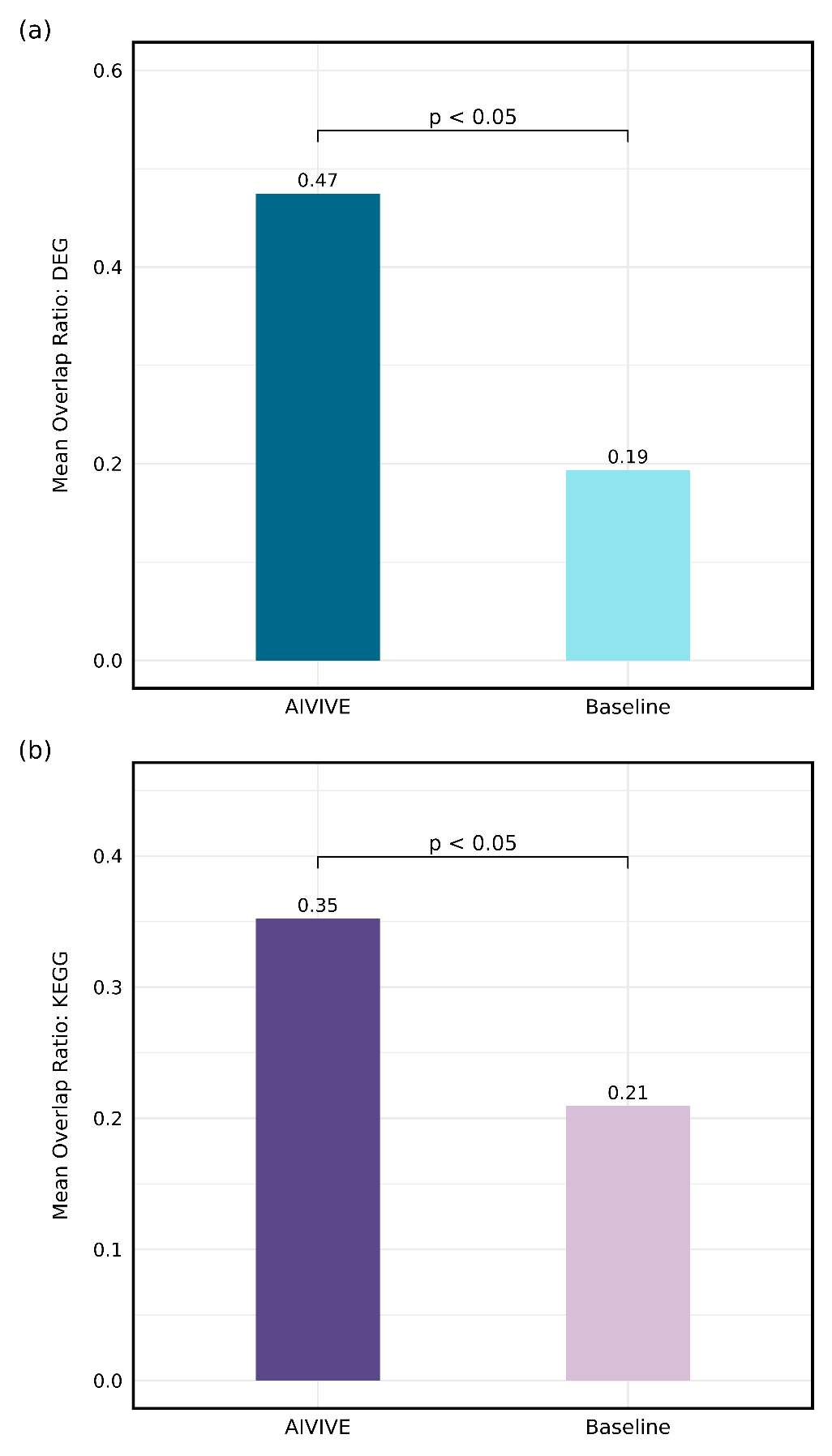


**Supplemental Figure 4:** *Comparison of DEG and KEGG Pathway Overlap between Baseline and AIVIVE)*

DEGs were identified in real *in vitro* profiles, as well as in real *in vivo* and synthetic *in vivo* profiles generated by AIVIVE, under the 24-hour time point for the test set compounds. Overlap was assessed for two groups: (1) **‘Baseline’**, which represents DEG overlap between real *in vitro* and real *in vivo* profiles, and (2) **‘AIVIVE’**, which represents DEG overlap between real *in vivo* and AIVIVE-generated *in vivo* profiles. A similar approach was applied for the KEGG pathway enrichment analysis.

Panel (a) shows a comparison of the average DEG overlap ratio between the AIVIVE and Baseline groups, while Panel (b) illustrates the comparison of the average KEGG pathway overlap ratio between the two groups.
